# Fabrication of Ultra-Fine Ag NPs on TiO_2_ Thin Films by Alcohol-Assisted Photodeposition Process for Photocatalysis-Related Applications

**DOI:** 10.3390/ma17061354

**Published:** 2024-03-15

**Authors:** Salih Veziroglu

**Affiliations:** 1 Chair for Multicomponent Materials, Department of Materials Science, Faculty of Engineering, Kiel University (CAU), Kaiserstr. 2, 24143 Kiel, Germany; sve@tf.uni-kiel.de; 2 Kiel Nano, Surface and Interface Science KiNSIS, Kiel University, Christian Albrechts-Platz 4, 24118 Kiel, Germany

**Keywords:** photocatalyst, TiO_2_, thin film, Ag nanoparticles, alcohols, photocatalytic deposition

## Abstract

Noble metal/semiconductor nanocomposites have been synthesized using various methods, including precipitation and hydrothermal and electrochemical processes. Among these, the photodeposition method stands out for its simplicity, without the need for high temperatures, redox agents, or complex steps. This method facilitates the control over noble metal nanoparticle size by adjusting parameters such as metal precursor concentration, irradiation time, and power. However, understanding the interaction between solid and liquid interfaces, particularly the role of solution viscosity in the growth process, remains a challenge. This knowledge is crucial for precise control over nanoparticle size and distribution. Our study highlights the influence of viscosity, manipulated through different alcohols, on the formation of Ag nanostructures on TiO_2_ thin films via photodeposition, offering insights into optimizing nanocomposite synthesis.

## 1. Introduction

Photocatalysis has proven to be a versatile approach with many uses, such as solar water splitting, CO_2_ reduction, self-purification, water purification, and sensing, and has attracted significant interest in recent decades [1,2,3,4,5]. There has been rapid progress in this field, particularly with the development of innovative photodeposited nanocomposite structures using the photodeposition process (also known as the photocatalytic deposition process) [6,7,8]. In this sophisticated process, the preparation of (photo)catalytic materials is advantageous because of their mild reaction conditions, simplicity, and eco-friendliness compared to more complex approaches [9,10,11]. Furthermore, light (usually ultraviolet (UV)) is introduced as a controllable factor in the production of precision nanostructures [12,13,14]. More recently, the variety of supports for photodeposition has been widened, ranging from semiconductors such as titanium (IV) oxide (TiO_2_) and zinc (II) oxide (ZnO) to carbon materials and metals, which exhibit localized surface plasmon resonance effects [8,15]. The techniques used to control the size, distribution, and morphology of photodeposited materials are becoming increasingly diverse and offer unique advantages in the creation of single atomic, epitaxial, and layered structures for specific applications [8,16,17].

Typically, the mechanism of photodeposition involves the absorption of light by a semiconductor (e.g., TiO_2_ and ZnO), resulting in the formation of electron-hole pairs. These carriers migrate to the semiconductor surface where they participate in redox reactions [17,18]. Reductive photodecomposition involves photoexcited electrons reducing metal ions (such as Au^3+^, Ag^+,^ and Pt^2+^) to their metallic forms (Au^0^, Ag^0^, and P^0^), while oxidative photodecomposition involves holes oxidizing a species, leading to the deposition of metal oxide [19,20,21,22]. The efficiency of this process is influenced by various factors, including the redox potential of the metals, the semiconductor’s band positions, the energy of the incoming photons, and the availability of active sites on the semiconductor surface [8]. For instance, longer exposure to light can boost the production of charge carriers, affecting the localized deposition process and leading to larger metal nanoparticles [23]. The introduction of sacrificial reagents can prevent the recombination of these carriers, enhancing their migration rate and the growth of nanoparticles. Additional elements such as Schottky junctions, adsorption, and competing reactions also play a role in the photodeposition process [24]. Semiconductors not only generate charge carriers but also act as a platform for the nucleation and growth of metal nanoparticles [25]. The formation of Schottky and ohmic junctions between the metal and semiconductor can promote effective electron transfer, concentrating photogenerated electrons at the nucleation sites [26]. To control photodeposition, it is crucial to manage the electron transfer rate and prevent electron clustering, which can otherwise result in uncontrolled particle growth [8]. Recent studies have aimed to thoroughly understand how solution chemistry, including the role of reducing agents and surfactants, affects the size, composition, and morphology of nanoparticles during photodeposition [27,28]. However, challenges remain in controlling nanoparticle formation due to the current limitations in real-time monitoring and a comprehensive understanding of nucleation and growth mechanisms in liquid environments [29].

In this study, our main objective is to examine the effect of the solution viscosity on the resulting ultra-fine Ag nanoparticles (NPs) on the TiO_2_ thin film surface by the photodeposition process. In our previous studies, we demonstrated how the decoration of TiO_2_ thin film with metallic Ag nanoparticles improves photocatalytic performance as well as sensing capability [30]. Thus, to achieve a high-quality (photo)catalyst, providing more insights into the growth mechanism of Ag NPs for better control is required. Such growth control could also be essential for the modification of plasmonic properties, which would be highly beneficial for visible-light-driven photocatalysis. For this purpose, we used five different solvents, water, methanol, ethanol, 1-propanol, and 1-butanol, to study the impact of viscosity. The composition and surface morphology of the fabricated Ag-TiO_2_ nanocomposites have been investigated by X-ray photoelectron spectroscopy (XPS) and scanning electron microscopy (SEM) analysis and discussed.

## 2. Materials and Methods

### 2.1. Fabrication of TiO_2_ Thin Films

The TiO_2_ thin films were deposited using reactive pulsed DC magnetron sputtering from a two-inch Ti target (Ti-Goodfellow, 99.99%, 5 cm diameter) with argon (Ar) as the process gas and oxygen (O_2_) as the reactive gas. Silicon wafer pieces (Si, 10 × 10 mm) served as the substrate. A base pressure of 10^−5^ Pa was achieved using a rotary pump (Agilent Technologies, Santa Clara, CA, USA, SH-110) and a turbo molecular pump (Pfeiffer Vacuum, Aßlar, Germany, HiPace 400). Before deposition, the Ti target was cleaned in pure Ar plasma for 15 min, followed by a 5 min conditioning phase with both Ar and O_2_ flow. The magnetron output power was set at 90 W, and the Ar: O_2_ ratio was 250 sccm—10 sccm during the deposition, controlled by two separate mass flow controllers (MKS MultiGas-Controller 647C), Andover, MA, USA. To ensure a uniform TiO_2_ film (thickness: 300 nm), the sample holder was rotated during deposition. Post-deposition, the TiO_2_ thin film samples were heat-treated for 1 h at 650 °C in an oven (Nabertherm, Lilienthal, Germany, LE 4/11/R6) and then air quenched to obtain crystalline and photocatalytically active surface [31].

### 2.2. Photodeposition of Ag NPs on TiO_2_ Thin Films

In a standard trial, 6.5 mL of silver nitrate (AgNO_3_ with a concentration of 1 × 10^−3^ M) was mixed with various alcohol media (methanol, ethanol, 1-propanol, and 1-butanol) in water using a UV-transparent quartz cuvette (Starna GmbH, Pfungstadt, Germany), as previously detailed [30]. Subsequently, TiO_2_ thin film samples were immersed in the cuvette and exposed to a low-intensity UV LED operating at a 365 nm wavelength and a power of 4.5 mWcm^−2^ for 1 and 5 min. The samples were then washed with deionized (DI) water to eliminate excess solution and allowed to air-dry at room temperature.

### 2.3. Materials Characterization

The prepared Ag-TiO_2_ thin films were analyzed using scanning electron microscopy (SEM, Supra55VP-Carl Zeiss, New York, NY, USA) to study their surface morphology and structure. The size and distribution of Ag NPs on the TiO_2_ thin film surface were investigated using the MATLAB-based MIPAR TM software (v4.1.0.5). Additionally, the chemical composition and surface chemistry of the sample were examined using X-ray photoelectron spectroscopy (XPS, Omicron Nano-Technology GmbH, Taunusstein, Germany, Al anode, 240 W), with the C1s peak at 284.8 eV serving as the calibration for all binding energies (BE) to account for the absorbed carbon on the sample surface.

## 3. Results and Discussion

### 3.1. Ag NPs Formation in Water Media

**Figure 1:**
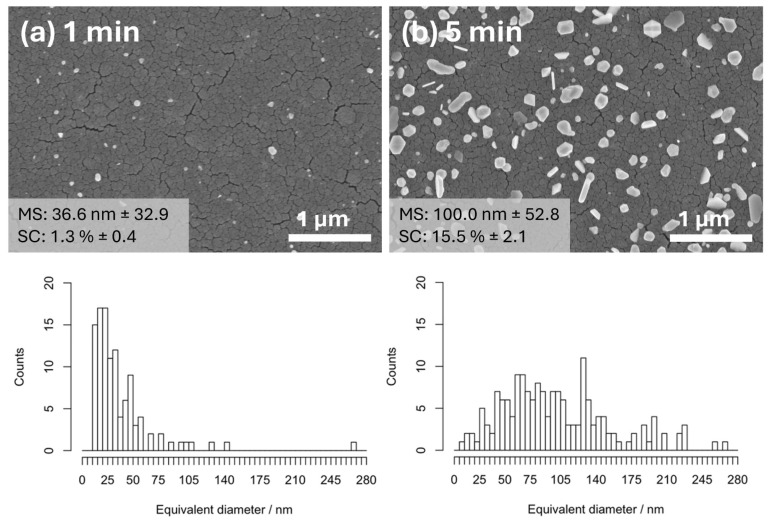
SEM images of Ag NPs on TiO_2_ thin film photodeposited in water media at (**a**) 1 min and (**b**) 5 min UV exposure with corresponding particle size distribution for each case. (MS: mean particle size and SC: surface coverage).

Figure 1 provides a detailed visual representation of the changes in surface morphology of silver nanoparticles (Ag NPs) photodeposited on titanium dioxide (TiO_2_) thin films, as observed at two distinct UV exposure times: 1 min and 5 min. The figure clearly shows that the average particle size and the degree of surface coverage of Ag NPs both show a marked increase when the duration of UV exposure is extended. This exponential growth in particle size and coverage is a direct consequence of the prolonged interaction with UV light. Furthermore, a notable enhancement in the primary particle size and the tendency for Ag NPs to agglomerate is evident with increased UV exposure. Specifically, after a 5 min exposure to UV light, the Ag NPs exhibit a threefold increase in particle size compared to their initial dimensions, while they exhibit ununiform morphology. Concurrently, the surface coverage of the TiO_2_ thin films with Ag NPs is observed to expand nearly twelvefold. This phenomenon can be attributed to the energy provided by the UV light, which facilitates the nucleation and growth processes of Ag NPs, leading to larger and more densely packed nanoparticle structures on the TiO_2_ thin film.

**Figure 2:**
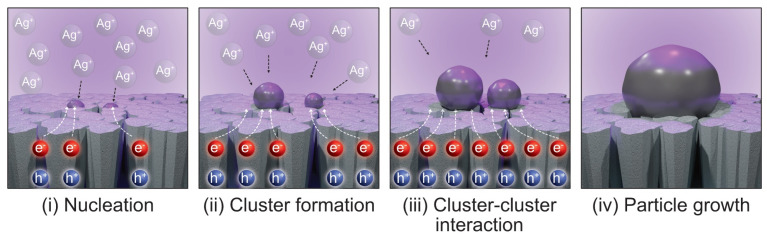
Schematic illustration of step-by-step growth of Ag nanoparticles on TiO_2_ thin film surface by photodeposition process.

In a typical explanation of the photodeposition process, UV illumination on the TiO_2_ surface initiates the creation of electron/hole pairs, which in turn produce free electrons (as schematically shown in Figure 2). These electrons are crucial for the reduction of Ag ions encountered by the surface, facilitating their accumulation as nanoparticles on the TiO_2_ surface (i: nucleation). As the process progresses, these nanoparticles serve as electron traps, enlarging by drawing in additional Ag ions (ii: cluster formation). This method is straightforward to implement and can be scaled up for larger samples (iii: cluster–cluster interaction and iv: particle growth). The characteristics of the Ag nanoparticles, including their size and spatial arrangement, are determined by factors such as the electron availability on the TiO_2_ surface, the Ag ion concentration in the solution, and the deposition duration. However, the process might be more complicated than such simple explanations. Therefore, the mechanism part will be detailed and explained in the following sections.

### 3.2. Ag NPs Formation in 25% Alcohol Media (Methanol, Ethanol, and 1-Propanol)

**Figure 3:**
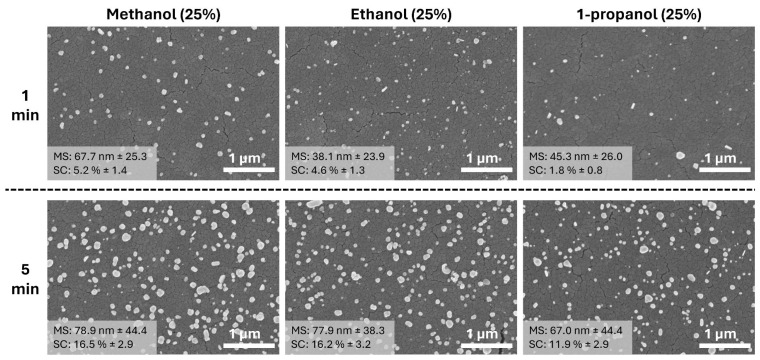
SEM images of Ag NPs on TiO_2_ thin film photodeposited in different 25% alcohol media (methanol, ethanol, and 1-propanol) at different UV exposures. (MS: mean particle size and SC: surface coverage). Detailed particle size distribution can be seen in Figure S1.

SEM analysis of Ag NPs on TiO_2_ thin film surfaces reveals interesting variations in particle size and surface coverage when different alcohols are used as media for photodeposition (Figure 3). When methanol, ethanol, and 1-propanol are employed, the mean sizes of the Ag NPs are 67.7 nm, 38.1 nm, and 45.3 nm, respectively. These sizes are slightly larger than those obtained when only water is used as the medium. Despite the differences in particle size, there is no clear correlation between the mean size of Ag NPs and the type of alcohol used during the short photodeposition time of 1 min. The surface coverage of Ag NPs is quite similar in methanol and ethanol, at 5.2% and 4.6%, respectively. However, in 1-propanol media, the coverage is significantly lower, at only 1.8%. After extending the photodeposition time to 5 min, both the surface coverage and mean size of the particles increase across all conditions. Notably, the particles photodeposited in methanol and ethanol media show nearly identical surface coverage (16.5% and 16.2%, respectively) and mean size (78.9 nm and 77.9 nm, respectively). By contrast, while the mean size of particles in 1-propanol remains similar to those in methanol and ethanol (67.0 nm), the surface coverage is relatively lower at 11.9%. These observations suggest that the choice of alcohol can influence the growth dynamics and distribution of Ag NPs on TiO_2_ surfaces during photodeposition, although the exact mechanisms behind these effects are not immediately apparent from the SEM data alone.

**Figure 4:**
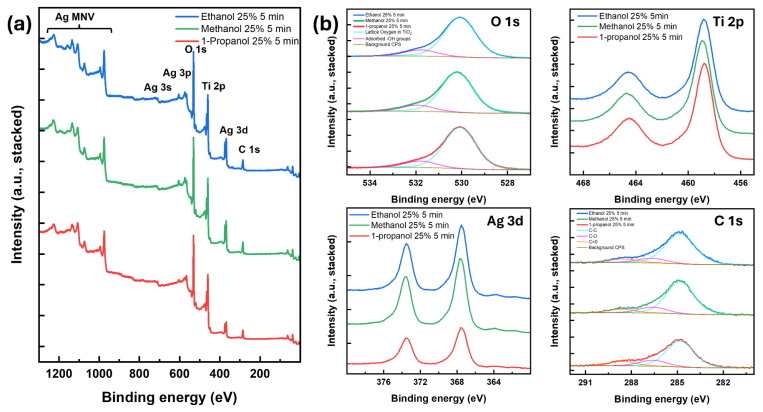
(**a**) Wide and (**b**) high resolution (O 1s, Ti 2p, Ag 3d, and C 1s) XPS spectra of Ag NPs on TiO_2_ thin film photodeposited in 25% alcohol media (methanol, ethanol, and 1-propanol) at 5 min. (In wide XPS spectra, the Y-axis is offset by 70% for better comparison).

The X-ray photoelectron spectroscopy (XPS) technique was employed to examine the chemical composition of the sample surfaces. The process involves directing X-rays at the sample, which dislodges electrons from the surface atoms. A detector then measures the energy of these electrons, which corresponds to the bonding energies of specific elements. The electron count is plotted against the bonding energy to generate the sample’s spectrum. The samples photodeposited in 25% alcohol for 5 min were studied, and their wide XPS spectra are displayed in Figure 4a. The analysis verified the presence of titanium, oxygen, silver, and carbon [32,33]. When the individual peaks are enlarged and the background is eliminated (as depicted in Figure 4b), the spectra of all samples appear nearly identical [34,35]. The Ag 3d spectrum contains two peaks which can be attributed to Ag 3d_5/2_ and Ag 3d_3/2_ spin-orbit coupling, respectively, which indicates the formation of metallic silver (Ag^0^) [36]. However, the Ag content is consistent in the methanol and ethanol samples, but less silver is found in the 1-propanol sample. This difference is attributed to the surface coverages of the particles, which are 16.5% and 16.2% on methanol and ethanol, respectively, but only 11.9% on the iso-propanol sample. A significant amount of C 1s was observed, likely due to adsorption on the surface from exposure of the samples to air or remnants from the alcohol molecules. Other differences become apparent when calculating the atomic % composition of the elemental components, as shown in Table S1. The TiO_2_ concentration remains nearly constant across all samples. However, there is a slight increase in oxygen across the samples, possibly due to overexposure of the samples to air. More specifically, the oxygen contribution (photodeposition in methanol, ethanol, and 1-propanol) can be divided into lattice and adsorbed oxygen, approximately 85% and 15%, respectively. The slight decrease in carbon seems counterintuitive because the carbon chain becomes longer from methanol to 1-propanol. This could be because the photoexcited electrons from TiO_2_ first partially reduced the alcohol molecules to H_2_O and CO_2_, giving them off as a gas, thereby reducing the amount of carbon available in the solution.

**Figure 5:**
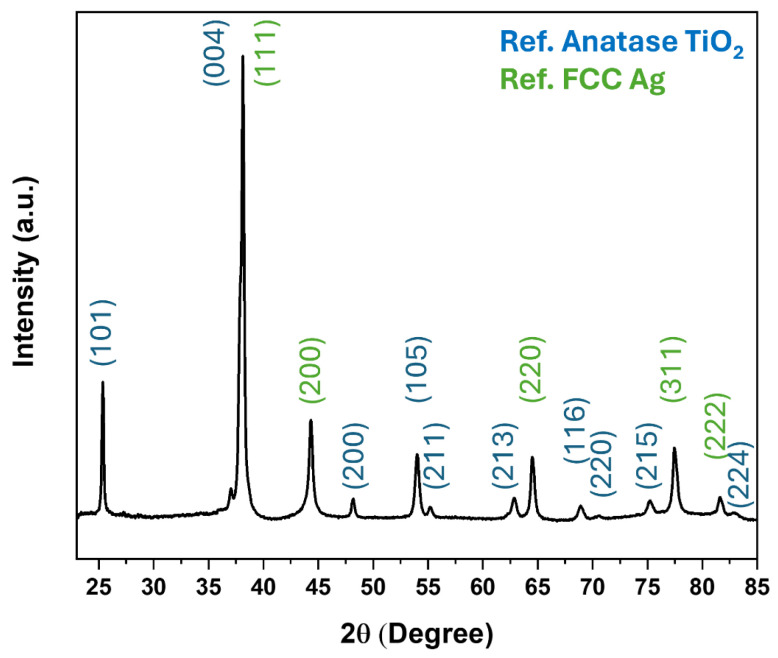
XRD pattern of Ag NPs on TiO_2_ thin film photodeposited in 25% methanol for 5 min.

The X-ray diffraction pattern of TiO_2_ thin film decorated with Ag NPs is shown in Figure 5. The XRD spectra revealed that the TiO_2_ thin film is crystallized, revealing XRD lines related to the anatase phase (JCPDS card no: 21-1272); no other phase (rutile or brookite) is detected. The decoration of anatase TiO_2_ thin films with FCC Ag nanoparticles introduces additional diffraction peaks corresponding to the Ag crystal structure. The XRD pattern of FCC Ag nanoparticles shows distinct peaks at 2θ positions around 38.09, 44.33, 64.43, 77.41, and 81.63, which correspond to the (111), (200), (220), (311), and (222) Miller indices, respectively. These peaks confirm the face-centered cubic crystalline structure of the Ag nanoparticles [37]. The intensity and sharpness of these peaks can indicate the degree of crystallinity and the size of the Ag nanoparticles.

### 3.3. Ag NPs Formation in 50% Alcohol Media (Methanol, Ethanol, and 1-Propanol)

**Figure 6:**
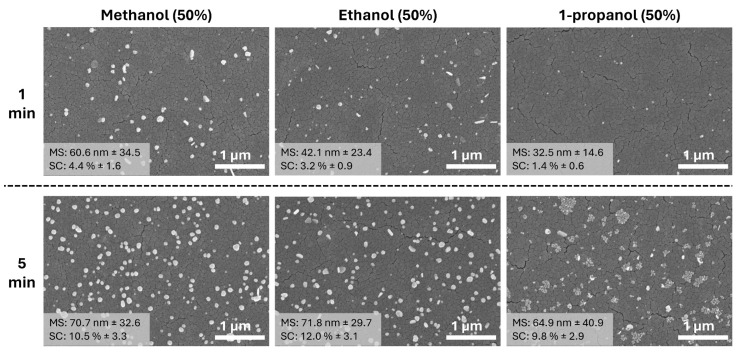
SEM images of Ag NPs on TiO_2_ thin film photodeposited in different 50% alcohol media (methanol, ethanol, and 1-propanol) at different UV exposures. (MS: mean particle size and SC: surface coverage). Detailed particle size distribution can be seen in Figure S2.

When the deposition time is 1 min, 50% and 25% of alcohol media show similar patterns in particle size and surface coverage as shown in Figure 6. The initial mean particle size and surface coverage decrease from 60.6 nm to 32.5 nm when methanol and 1-propanol solutions are used, respectively. One can easily see some ununiform structures, especially in the 1 min deposition. However, after 5 min of photodeposition, the surface coverage of the particles significantly decreases compared to deposition under 25% alcohol media. This suggests that the duration of photodeposition and the concentration of the alcohol media can significantly influence the surface coverage of Ag NPs on the TiO_2_ thin film. Despite the decrease in surface coverage, the particle size remains nearly similar under 50% alcohol media (methanol, ethanol, and 1-propanol), with sizes of 70.7, 71.8, and 64.9 nm, respectively. This indicates that the particle size is less affected by the changes in alcohol media concentration and photodeposition time.

### 3.4. Ag NPs Formation in 100% Alcohol Media (Methanol, Ethanol, and 1-Propanol)

**Figure 7:**
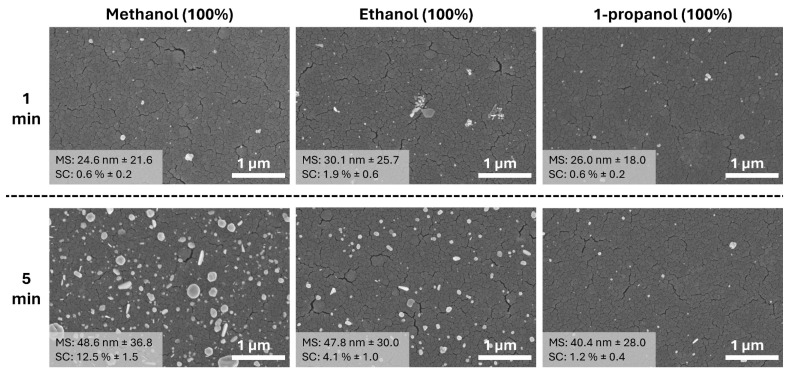
SEM images of Ag NPs on TiO_2_ thin film photodeposited in different 100% alcohol media (methanol, ethanol, and 1-propanol) at different UV exposures. (MS: mean particle size and SC: surface coverage). Detailed particle size distribution can be seen in Figure S3.

It was observed that an increase in alcohol concentration leads to a reduction in the mean size of primary Ag NPs, with sizes averaging around 25 nm, which are the smallest particles among the others (Figure 7). This suggests that alcohol acts as a moderating agent, influencing the nucleation and growth rates of the nanoparticles. However, the early stages of growth present a challenge for surface coverage analysis due to the non-uniform structure of the nanoparticles. Furthermore, as the deposition time extends from 1 min to 5 min, there is a noticeable increase in particle size, indicating that longer deposition times promote particle growth. Interestingly, the size increment is less pronounced in media containing 25% and 50% alcohol, which could be attributed to the alcohol’s role in stabilizing the nanoparticles and preventing excessive growth. A surprising outcome of the SEM analysis is the drastic increase in surface coverage of Ag NPs, which jumps from 0.6% to 12.5% when using 100% methanol as the reaction medium. This substantial increase suggests that methanol affects the nucleation and growth of Ag NPs and significantly enhances their distribution across the TiO_2_ thin film surface.

### 3.5. Interfacial Charge Transfer Processes

**Figure 8:**
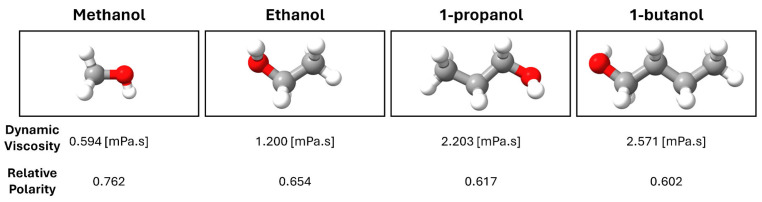
Dynamic viscosity and relative polarity values of alcohols at around 25 °C.

Photodeposition is a process where light is used to reduce metal ions to their metallic form, which are then deposited onto a substrate like TiO_2_. The choice of solvent can significantly influence the photodeposition process due to some factors (Figure 8). The polarity of the solvent can affect the reduction potential of the metal ions and the interaction between the solvent and both the substrate and the metal ions. This can influence the nucleation and growth rates of nanoparticles. For example, higher viscosity can slow down the diffusion of metal ions toward the substrate, potentially leading to smaller and more uniformly distributed nanoparticles. Here, methanol can act as a reducing agent and a source of protons, which might influence the charge balance on the TiO_2_ surface and potentially affect the size of Ag nanoparticles by altering the surface chemistry and the reduction process. Methanol’s relatively low viscosity and moderate polarity also facilitate the diffusion of Ag ions towards the TiO_2_ surface. Like methanol, ethanol can also act as a solvent and reducing agent but with slightly different chemical properties due to its larger molecular size and different functional groups. The effect of ethanol on the size of Ag nanoparticles would likely be like that of methanol, with potential differences arising from its slightly higher viscosity and different reactivity. 1-propanol has a higher viscosity and is more sterically hindered than methanol and ethanol, which could affect the diffusion of Ag ions and the nucleation process on the TiO_2_ surface. This might lead to differences in the size and distribution of Ag nanoparticles compared to methanol and ethanol.

The kinetics of photodeposition are directly related to the rates at which the reactants are transported to the TiO_2_ surface and the subsequent reactions that occur there. Higher viscosity solvents typically have lower diffusion coefficients, which can slow down the transport of metal ions to the TiO_2_ surface, leading to slower photodeposition rates. Conversely, lower-viscosity solvents would facilitate faster diffusion and potentially increase the rate of photodeposition. Solvent viscosity can influence the uniformity and density of the metal nanoparticles on the TiO_2_ surface. In a high-viscosity solvent, the slower diffusion may result in less uniform coverage and potentially larger, but fewer, metal nanoparticles due to the longer time required for ions to reach the surface. By contrast, a low-viscosity solvent might promote a more uniform and dense coverage of smaller nanoparticles due to the more efficient transport of ions. Therefore, it is expected that using 100% 1-butanol could be considered to synthesize ultra-fine Ag NPs on the thin film surface.

### 3.6. Ag NPs Formation in 100% 1-Butanol

**Figure 9:**
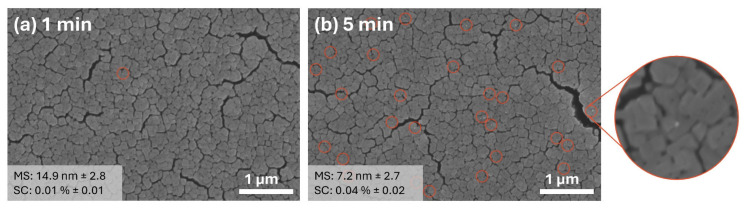
SEM images of Ag NPs on TiO_2_ thin film photodeposited in 100% 1-butanol media at (**a**) 1 min and (**b**) 5 min UV exposure. Red circles represent individual Ag NPs on the TiO_2_ thin film surface. (MS: mean particle size and SC: surface coverage).

The 1-butanol, having the highest viscosity among the solvents considered (methanol, ethanol, and 1-propanol), is expected to influence the size and distribution of Ag NPs on TiO_2_ surfaces. This high viscosity is anticipated to result in the formation of smaller Ag NPs due to the slower diffusion rates of silver ions in the solvent, allowing for more controlled growth of the nanoparticles. Indeed, results confirm that the viscosity of the solvent plays a significant role in determining the size and surface coverage of the nanoparticles as shown in Figure 9. After a 5 min deposition period, the smallest Ag nanoparticles, with an average size of 7.2 nm, were synthesized on TiO_2_ in a solvent environment with 100% 1-butanol content. However, this process presents challenges in achieving proper surface coverage without increasing nanoparticle agglomeration. The difficulty lies in balancing the conditions to optimize the distribution and size of Ag NPs while minimizing their tendency to clump together.

## 4. Conclusions

The development of ultra-fine Ag nanoparticles on TiO_2_ thin film surfaces has emerged as an innovative advancement in the field of photocatalysis, offering significant potential for applications in solar water splitting, self-cleaning surfaces, and CO_2_ reduction. Our research has focused on the photodeposition method to synthesize Ag/TiO_2_ nanocomposites, a technique that allows for precise control over the deposition of Ag NPs on TiO_2_ thin film substrates. By accurately adjusting the viscosity of the photodeposition solution (by using methanol, ethanol, 1-propanol, and 1-butanol), we have been able to modulate the photocatalytic reduction rate of Ag^+^ ions (mainly mobility of the ions), a critical factor that influences the morphology and distribution of Ag NPs on the TiO_2_ thin film surface. Our findings reveal that the viscosity of the solution plays a pivotal role in the growth dynamics of Ag NPs, affecting both their size and shape. A higher solution viscosity might tend to slow down the mobility of Ag^+^ ions at the TiO_2_ interface, favoring a growth mechanism characterized by the sequential addition of atoms or small particles. This process promotes the formation of smaller and more uniformly distributed particles on the surface.The use of 1-butanol as a solvent in our experiments has demonstrated the feasibility of producing ultra-fine Ag NPs without the need for complex equipment typically associated with high vacuum techniques. The ability to control the size and distribution of Ag NPs through the adjustment of solution viscosity allows us to optimize the photocatalytic performance of Ag/TiO_2_ nanocomposites. These advancements hold promise for addressing some of the most pressing environmental challenges of our time, including sustainable energy production, pollution mitigation, and CO_2_ reduction.

## Data Availability

Data are contained within the article.

## Supplementary Materials

The following supporting information can be downloaded at https://www.mdpi.com/article/10.3390/ma17061354/s1, Figure S1: The particle size distribution histogram of Ag NPs on TiO_2_ thin film photodeposited in different 25% alcohol media (methanol, ethanol, and 1-propanol) at different UV exposures.; Figure S2: The particle size distribution histogram of Ag NPs on TiO_2_ thin film photodeposited in different 50% alcohol media (methanol, ethanol, and 1-propanol) at different UV exposures.; Figure S3: The particle size distribution histogram of Ag NPs on TiO_2_ thin film photodeposited in different 100% alcohol media (methanol, ethanol, and 1-propanol) at different UV exposures.; Table S1: The chemical composition of the prepared samples in different 50% alcohol media (methanol, ethanol, and 1-propanol) for 5 min.

## References

[1] Waiskopf N., Ben-Shahar Y., Banin U. (2018). Photocatalytic Hybrid Semiconductor–Metal Nanoparticles; from Synergistic Properties to Emerging Applications. Adv. Mater..

[2] Ahmed S.N., Haider W. (2018). Heterogeneous Photocatalysis and Its Potential Applications in Water and Wastewater Treatment: A Review. Nanotechnology.

[3] Fang S., Rahaman M., Bharti J., Reisner E., Robert M., Ozin G.A., Hu Y.H. (2023). Photocatalytic CO_2_ Reduction. Nat. Rev. Methods Primers.

[4] Tao X., Zhao Y., Wang S., Li C., Li R. (2022). Recent Advances and Perspectives for Solar-Driven Water Splitting Using Particulate Photocatalysts. Chem. Soc. Rev..

[5] Veziroglu S., Röder K., Gronenberg O., Vahl A., Polonskyi O., Strunskus T., Rubahn H.-G., Kienle L., Adam J., Fiutowski J. (2019). Cauliflower-like CeO_2_–TiO_2_ Hybrid Nanostructures with Extreme Photocatalytic and Self-Cleaning Properties. Nanoscale.

[6] Bhuskute B.D., Ali-Löytty H., Honkanen M., Salminen T., Valden M. (2022). Influence of the Photodeposition Sequence on the Photocatalytic Activity of Plasmonic Ag–Au/TiO_2_ Nanocomposites. Nanoscale Adv..

[7] Lee Y., Kim T., Kim B., Choi S., Kim K. (2023). Synthesis of TiO_2_/MoSx/Ag Nanocomposites via Photodeposition for Enhanced Photocatalysis and Membrane Fouling Mitigation. J. Environ. Chem. Eng..

[8] Wenderich K., Mul G. (2016). Methods, Mechanism, and Applications of Photodeposition in Photocatalysis: A Review. Chem. Rev..

[9] Wang L., Porto C.L., Palumbo F., Modic M., Cvelbar U., Ghobeira R., De Geyter N., De Vrieze M., Kos Š., Serša G. (2021). Synthesis of Antibacterial Composite Coating Containing Nanocapsules in an Atmospheric Pressure Plasma. Mater. Sci. Eng. C.

[10] Bai Q., Shupyk I., Vauriot L., Majimel J., Labrugere C., Delville M.-H., Delville J.-P. (2021). Design of Metal@Titanium Oxide Nano-Heterodimers by Laser-Driven Photodeposition: Growth Mechanism and Modeling. ACS Nano.

[11] Liu H., Battiato S., Pellegrino A.L., Paoli P., Rossi P., Jiménez C., Malandrino G., Muñoz-Rojas D. (2017). Deposition of Metallic Silver Coatings by Aerosol Assisted MOCVD Using Two New Silver β-Diketonate Adduct Metalorganic Precursors. Dalton Trans..

[12] Bhardwaj S., Sharma D., Kumari P., Pal B. (2020). Influence of Photodeposition Time and Loading Amount of Ag Co-Catalyst on Growth, Distribution and Photocatalytic Properties of Ag@TiO_2_ Nanocatalysts. Opt. Mater..

[13] Zhao H., Mao Q., Jian L., Dong Y., Zhu Y. (2022). Photodeposition of Earth-Abundant Cocatalysts in Photocatalytic Water Splitting: Methods, Functions, and Mechanisms. Chin. J. Catal..

[14] Fresno F., Portela R., Suárez S., Coronado J.M. (2014). Photocatalytic Materials: Recent Achievements and near Future Trends. J. Mater. Chem. A.

[15] O’Rourke C., Wells N., Mills A. (2019). Photodeposition of Metals from Inks and Their Application in Photocatalysis. Catal. Today.

[16] Zhao F., Bai Q., Xia C., Hao J., Gayot M., Delville J.-P., Delville M.-H. (2023). Core–Shell Nanoheterodimers: Laser-Assisted Deposition of Single Bimetallic Au@M (M = Au, Ag, Pd, Pt) Nanodots on TiO_2_ Nanoparticles. Mater. Adv..

[17] Lee Y., Kim E., Park Y., Kim J., Ryu W., Rho J., Kim K. (2018). Photodeposited Metal-Semiconductor Nanocomposites and Their Applications. J. Mater..

[18] Chan S.C., Barteau M.A. (2005). Preparation of Highly Uniform Ag/TiO_2_ and Au/TiO_2_ Supported Nanoparticle Catalysts by Photodeposition. Langmuir.

[19] Li Y., Yang L., He H., Sun L., Wang H., Fang X., Zhao Y., Zheng D., Qi Y., Li Z. (2022). In Situ Photodeposition of Platinum Clusters on a Covalent Organic Framework for Photocatalytic Hydrogen Production. Nat. Commun..

[20] Veziroglu S., Ghori M.Z., Kamp M., Kienle L., Rubahn H.-G., Strunskus T., Fiutowski J., Adam J., Faupel F., Aktas O.C. (2018). Photocatalytic Growth of Hierarchical Au Needle Clusters on Highly Active TiO_2_ Thin Film. Adv. Mater. Interfaces.

[21] Kong J., Qin Y.-H., Wang T.-L., Wang C.-W. (2020). Photodeposition of Pt Nanoparticles onto TiO_2_@CNT as High-Performance Electrocatalyst for Oxygen Reduction Reaction. Int. J. Hydrogen Energy.

[22] Shondo J., Veziroglu S., Tjardts T., Fiutowski J., Schröder S., Mishra Y.K., Strunskus T., Rubahn H., Faupel F., Aktas O.C. (2022). Selective Adsorption and Photocatalytic Clean-Up of Oil by TiO_2_ Thin Film Decorated with p-V_3_ D_3_ Modified Flowerlike Ag Nanoplates. Adv. Mater. Interfaces.

[23] Veziroglu S., Obermann A.-L., Ullrich M., Hussain M., Kamp M., Kienle L., Leißner T., Rubahn H.-G., Polonskyi O., Strunskus T. (2020). Photodeposition of Au Nanoclusters for Enhanced Photocatalytic Dye Degradation over TiO_2_ Thin Film. ACS Appl. Mater. Interfaces.

[24] Majeed I., Ali H., Idrees A., Arif A., Ashraf W., Rasul S., Khan M.A., Nadeem M.A., Nadeem M.A. (2022). Understanding the Role of Metal Supported on TiO_2_ in Photoreforming of Oxygenates. Energy Adv..

[25] Chen G., Li R., Huang L. (2023). Advances in Photochemical Deposition for Controllable Synthesis of Heterogeneous Catalysts. Nanoscale.

[26] Yan F., Wang Y., Zhang J., Lin Z., Zheng J., Huang F. (2014). Schottky or Ohmic Metal–Semiconductor Contact: Influence on Photocatalytic Efficiency of Ag/ZnO and Pt/ZnO Model Systems. ChemSusChem.

[27] Ferrah D., Tieu P. (2020). Controllable Growth of Copper on TiO_2_ Nanoparticles by Photodeposition Based on Coupled Effects of Solution Viscosity and Photoreduction Rate for Catalysis-Related Applications. ACS Appl. Nano Mater..

[28] Lisiecki I. (2005). Size, Shape, and Structural Control of Metallic Nanocrystals. J. Phys. Chem. B.

[29] You H., Fang J. (2016). Particle-Mediated Nucleation and Growth of Solution-Synthesized Metal Nanocrystals: A New Story beyond the LaMer Curve. Nano Today.

[30] Shondo J., Veziroglu S., Tjardts T., Sarwar T.B., Mishra Y.K., Faupel F., Aktas O.C. (2022). Nanoscale Synergetic Effects on Ag–TiO_2_ Hybrid Substrate for Photoinduced Enhanced Raman Spectroscopy (PIERS) with Ultra-Sensitivity and Reusability. Small.

[31] Vahl A., Veziroglu S., Henkel B., Strunskus T., Polonskyi O., Aktas O.C., Faupel F. (2019). Pathways to Tailor Photocatalytic Performance of TiO_2_ Thin Films Deposited by Reactive Magnetron Sputtering. Materials.

[32] Daniel L., Nagai H., Yoshida N., Sato M. (2013). Photocatalytic Activity of Vis-Responsive Ag-Nanoparticles/TiO_2_ Composite Thin Films Fabricated by Molecular Precursor Method (MPM). Catalysts.

[33] Ramasamy P., Seo D.-M., Kim S.-H., Kim J. (2012). Effects of TiO_2_ Shells on Optical and Thermal Properties of Silver Nanowires. J. Mater. Chem..

[34] Sahyun M.R.V., Serpone N. (1997). Primary Events in the Photocatalytic Deposition of Silver on Nanoparticulate TiO_2_. Langmuir.

[35] Zhao S., Cheng Z., Kang L., Li M., Gao Z. (2017). The Facile Preparation of Ag Decorated TiO_2_/ZnO Nanotubes and Their Potent Photocatalytic Degradation Efficiency. RSC Adv..

[36] Li S., Hu J., Yang Y., Zhao L., Qiao Y., Liu W., Liu P., Chen M. (2017). Ag/Nano-TiO_2_ Composite Compact Film for Enhanced Performance of Perovskite Solar Cells Based on Carbon Counter Electrodes. Appl. Phys. A.

[37] Ali M.H., Azad M.A.K., Khan K.A., Rahman M.O., Chakma U., Kumer A. (2023). Analysis of Crystallographic Structures and Properties of Silver Nanoparticles Synthesized Using PKL Extract and Nanoscale Characterization Techniques. ACS Omega.

