# Supplementary material for: Fabrication of Ultra-Fine Ag NPs on TiO2 Thin Films by Alcohol-Assisted Photodeposition Process for Photocatalysis-Related Applications
Source: Materials (Basel). 2024 Mar 15;17(6):1354. doi: 10.3390/ma17061354 (PMC10972172; doi:10.3390/ma17061354)
Supplement: Supplementary file 1 [file materials-17-01354-s001.zip › materials-2906524-supplementary.pdf]

Supplementary Information

Fabrication of Ultra-Fine Ag NPs on TiO_2 Thin Films by Alcohol-Assisted Photodeposition Process for Photocatalysis-Related Applications

Salih Veziroglu [1, 2]*

[1] Chair for Multicomponent Materials, Department of Materials Science, Faculty of Engineering, Kiel University (CAU), Kaiserstr. 2, 24143 Kiel, Germany

[2] Kiel Nano, Surface and Interface Science KiNSIS, Kiel University, Christian Albrechts-Platz 4 24118, Kiel, Germany

*Correspondence: sve@tf.uni-kiel.de

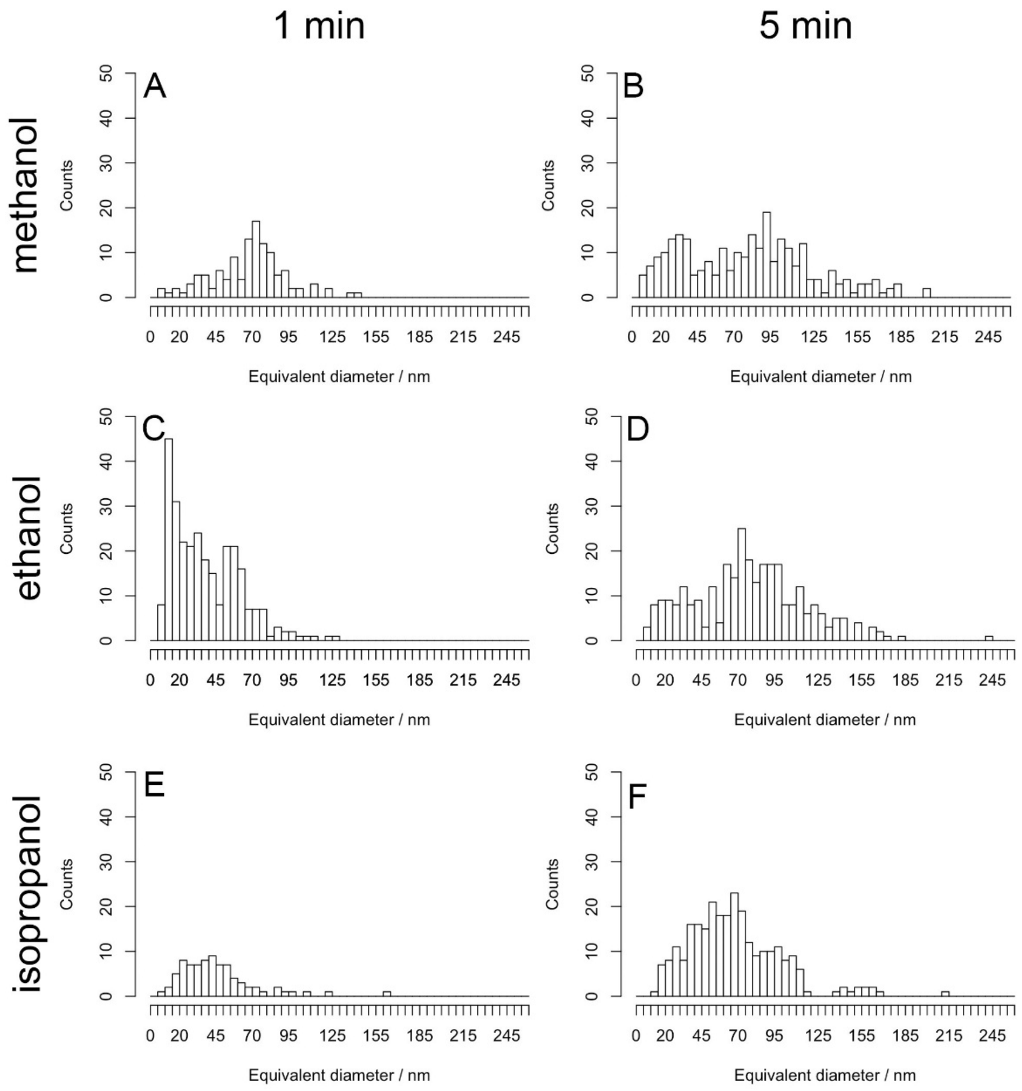

Figure S1. The particle size distribution histogram of Ag NPs on TiO_2 thin film photodeposited in different 25% alcohol media (methanol, ethanol, and 1-propanol) at different UV exposures.

Table S1. The chemical composition of the prepared samples in different 50% alcohol media (methanol, ethanol, and 1-propanol) for 5 minutes.

Photodeposition Media	Atomic Composition (%)			
	O 1s	C 1s	Ag 3d	Ti 2p
Methanol	51.98	24.32	4.91	18.79
Ethanol	53.92	23.26	4.47	18.35
1-propanol	56.15	21.77	2.88	19.20

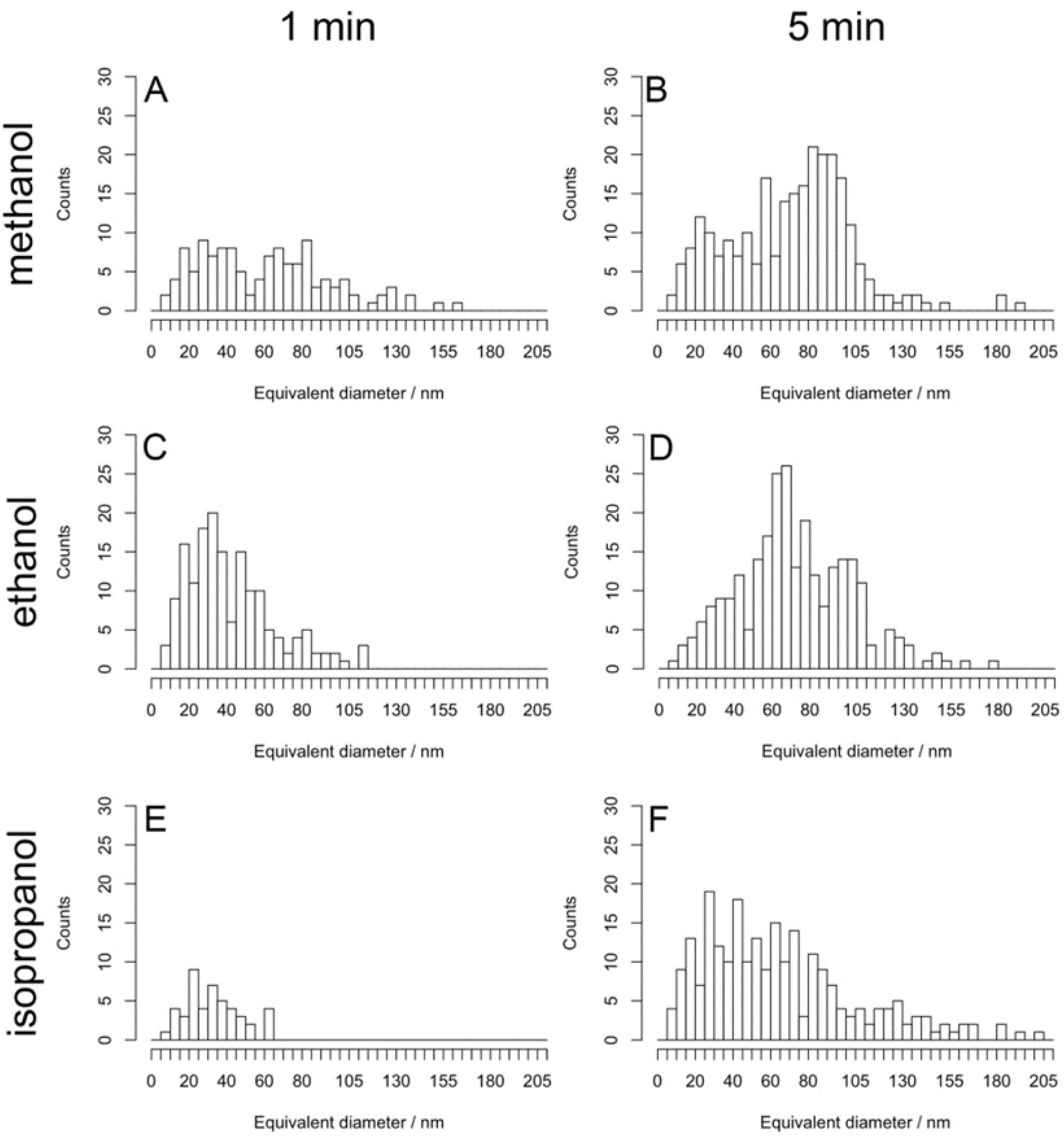

Figure S2. The particle size distribution histogram of Ag NPs on TiO_2 thin film photodeposited in different 50% alcohol media (methanol, ethanol, and 1-propanol) at different UV exposures.

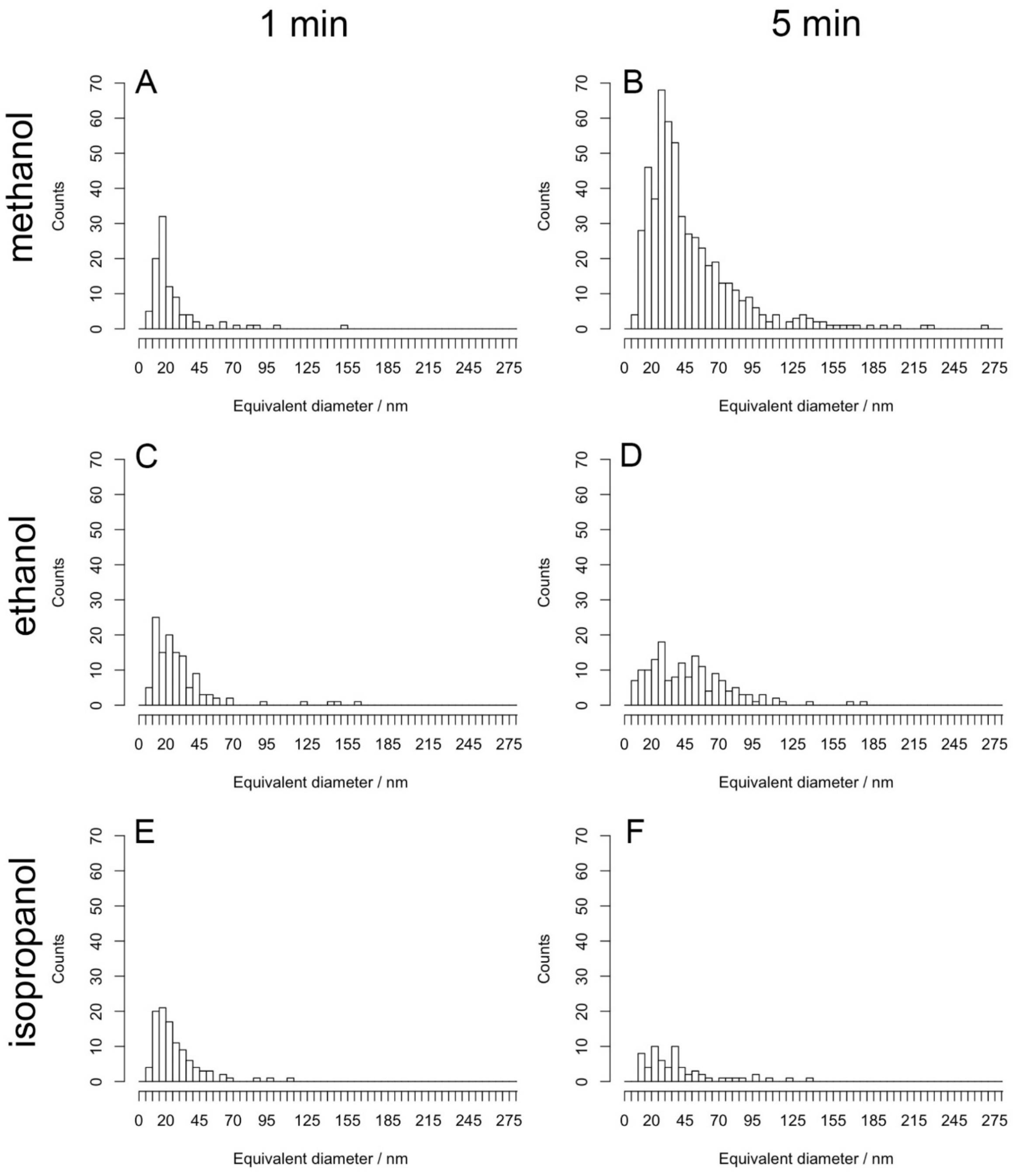

Figure S3. The particle size distribution histogram of Ag NPs on TiO_2 thin film photodeposited in different 100% alcohol media (methanol, ethanol, and 1-propanol) at different UV exposures.
